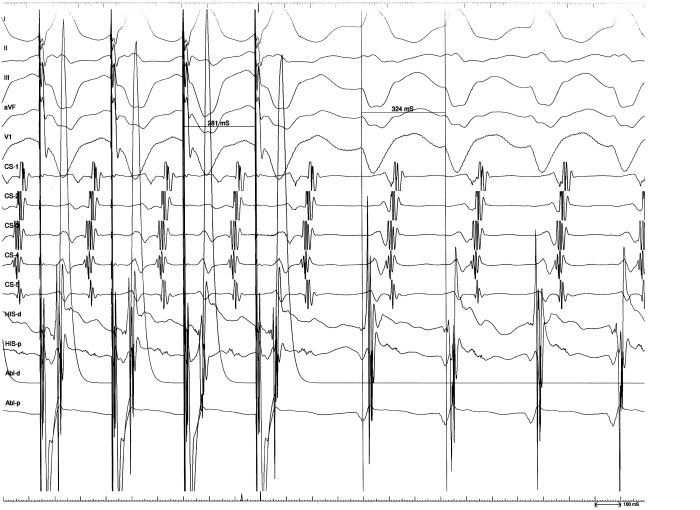# Ablating The Ventricular Insertion Of Atrio-fascicular Mahaim Fiber: Could Be Performed Safely?

**Published:** 2009-03-15

**Authors:** Valentino Ducceschi, Raffaele Vitale, Ewa Anna Sokola, Luca Ottaviano, Raffaele Sangiuolo, Giovanni Gregorio

**Affiliations:** 1 Department of Cardiology, San Luca Hospital, Vallo della Lucania (SA), Italy; 2 Department of Cardiology Fatebenefratelli Hospital, Naples, Italy

**Keywords:** atrio-fascicular accessory pathways, Mahaim pathway

## Abstract

We report a patient who underwent radiofrequency ablation of the distal insertion of an atrio-fascicular accessory pathway with decremental properties because of inability to map a suitable potential alongside the tricuspid annulus. Small, discrete potentials resembling those of Purkinje fiber were found at right ventricular apex. All these potentials showed early activation during tachycardia preceding the QRS onset of various degree. Pace mapping helped to localize the presumed main distal insertion of the atrio-fascicular accessory pathway in a region where damage of the His-purkinje system may ensue. This case report describes catheter ablation of an atriofascicular accessory pathway by targeting its distal (ventricular) insertion site.

## Introduction

Atrio-fascicular accessory pathways (AP) with decremental properties (i.e. Mahaim fibers) are associated with uncommon types of wide QRS complex tachycardia [1-5]. In many patients discrete, sharp potentials can be usually recognized along the lateral and posterior aspect of the tricuspid annulus and are considered the electrophysiologic counterpart of these anomalous connections [6-10]. Although these sites have been addressed as optimal sites for performing radiofrequency (RF) delivery in order to eliminate the conduction over the AP, there are many circumstances where it is reasonable to target the ventricular insertion of the AP. The most common of these circumstances is the inability to record a clear potential along the entire perimeter of the tricuspid annulus. This case report describes catheter ablation of an atriofascicular accessory pathway by targeting its distal (ventricular) insertion site.

## Case Report

A 44 years old male with structurally normal heart was referred to our institution because of long standing episodes of palpitation. Despite a history of sustained episodes of palpitation no evidence of arrhythmia was previously documented. The standard 12 leads EKG showed no baseline abnormality. The presence of structural heart disease was excluded by physical examination and trans thoracic echocardiography.After written informed consent was obtained an invasive EP study was performed in un-sedated, drug free state. One quadripolar and two decapolar diagnostic catheters were inserted through the right femoral and left subclavian veins and positioned respectively in the his bundle (HB) region, coronary sinus (CS) and  along the lateral wall of the right atrium. During the study the decapolar catheter located in the right atrium was moved to the right ventricular apex in order to record right bundle branch (RBB) potentials.

**Figure 1:**
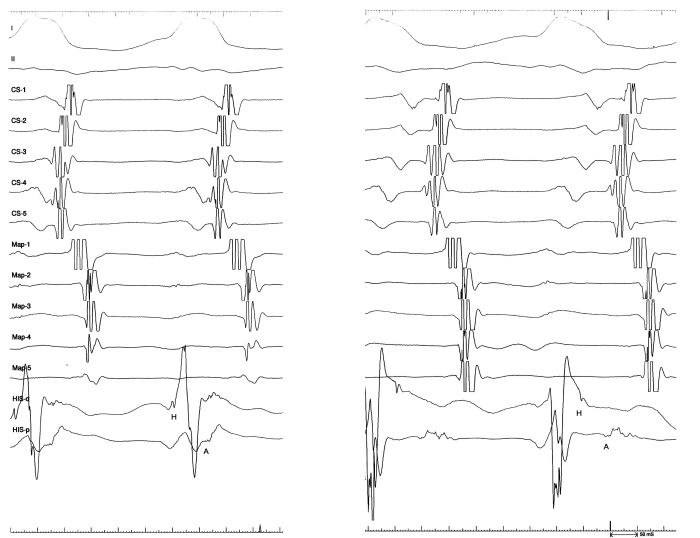


 During incremental atrial pacing, a progressive lengthening of the atrio-ventricular (A-V) interval was noted as well as progressive widening of the QRS and proportional shortening of the His-ventricular (H-V) interval.  A wide complex tachycardia exhibiting left bundle branch block (LBBB) morphology and superior axis was induced during pacing at 240 milliseconds (msec) from the high right atrium. The appearance of LBBB morphology was invariably associated with the progressive shortening of the HV interval up to the merging of the His potential with the local ventricular electrogram. After a stable RBB potential recording was obtained it was shown an inversion of the physiologic HB–RBB activation sequence during tachycardia, the HB resulting activated retrogradely. This tachycardia was characterized by an A:V ratio of 1:1 and a concentric retrograde atrial activation pattern (i.e. the earliest retrograde atrial activation was found at the His bundle region). Programmed atrial stimulation at different drive cycles (600-400 msec.) with double and triple extrastimuli successfully initiated tachycardia at coupling interval of less than 300 msec. The arrhythmia onset was always preceded by the occurrence of LBBB aberration of the last paced beat. During the recording session tachycardia cycle length (TCL) varied from 290 msec to 340 msec. These TCL variations were associated to proportional prolongations of V-A interval and QRS duration while the atrial retrograde activation sequence remained unmodified. Despite TCL lengthening and VA prolongation the HA interval remained constant. (Figure 1)

Atrial pacing at cycle length shorter than the TCL up to 240 msec successfully entrained the tachycardia without obtaining sinus rhythm restoration after pacing interruption. Rapid atrial pacing during sinus rhythm also initiated a self-limiting typical counterclockwise atrial flutter with 2:1 A-V pre-excited response. Ventricular pacing from the parahissian region at a cycle length just shorter than TCL invariably interrupted the tachycardia after His bundle capture was obtained.

**Figure 2:**
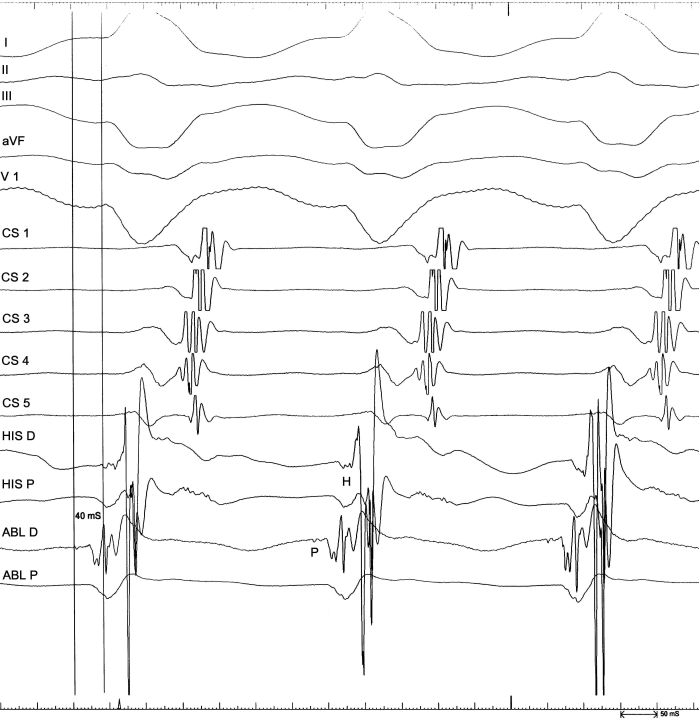


 The retrograde atrial activation in the His bundle trace always preceded the others and the atrial activation sequence remained unmodified during the entire recording session even in the presence of  TCL changes, this behavior was consistent with true antidromic AV reciprocating tachycardia using the Mahaim fascicle as antegrade limb. V-A prolongation without H-A modification excluded the involvement of a septal concealed accessory pathway as retrograde limb and also ruled out an atrioventriclar nodal reentrant tachycardia with a Mahaim fascicle participation as an innocent "bystander". We may speculate that this behavior probably reflected paroxysmal prolongation of the conduction time or block thorough the RBB next to the insertion of the Mahaim fiber.The ablation procedure was performed using a 7 F 4 mm ablation catheter (Blazer II, Boston Scientific). A careful and complete tricuspid ring mapping was performed in order to record a discrete potential resembling that recorded on His region, the so called Mahaim potential (MP). We failed to identify a discrete potential alongside the entire annulus so we decided to target the ventricular insertion. Given the superior axis of the arrhythmia we deliberately started from the right ventricular apex. Small, discrete potentials resembling those of Purkinje fiber were found near the right ventricular apex, these potentials showed various degrees of early activation during tachycardia and all preceded the His bundle activation. We delivered RF at a site with the apparently most negative local potential-QRS interval but we failed to terminate the arrhythmia. In order to reduce the number of unnecessary RF pulses we decided to perform pace mapping in a region where different sites with early local ventricular activation could be recorded. Pace mapping performed from the recording site of a small potential (SP), presumably the distal RBB, located 35 msec before the tachycardia QRS onset and 40 msec before the HB retrograde activation showed a QRS morphology identical to that observed during tachycardia. (Figures 2,3).

During sinus rhythm, pacing from this site was also followed by tachycardia initiation and of note the stim-QRS interval resembled the SP-QRS interval during tachycardia. This behavior was consistent with the antegrade limb distal insertion of the macroreentrant circuit. A single pulse of RF (50W, 60 °C) interrupted the tachycardia after less than 3 sec, the RF delivery was then continued for 60 seconds in order to achieve a more complete lesion. At the end of the procedure the arrhythmia was no longer inducible, incremental atrial pacing demonstrated normal AV conduction and an incomplete RBB block was evident. Patient was followed up for one year and there were no recurrences of tachycardia.

## Discussion

Recently unsuccessfull tricuspid annulus mapping for Mahaim discrete potential has been reported to be up to nearly 50 % [11]. When the proximal insertion of Mahaim AP cannot be clearly identified, the ventricular insertion should be targeted for RF ablation once the arrhythmia mechanism has been assessed. In order to obtain an optimal target for RF ablation, it might be worthwhile utilizing multiple selection criteria. Sites of recording a small, rapid early activated potentials nearby the right ventricular apex might be considered potentially successful targets for RF ablation of atrio-fascicular pathways. Because a wide distal branching of the Mahaim fiber may take place at least in some patients, the recognition of the main distal insertion may be troublesome and the selection of the optimal site for RF delivery may require additional criteria. Pace mapping can be an useful tool to identify potentially successful ablation site in a region of increased risk for RBB damage. The largest amount of local anticipation should be sought in order to obtain a reasonable probability of eliminating the arrhythmic substrate with the least number of RF pulses. An exact pace mapping can give additional information locating the distal insertion of the AP allowing to avoid unnecessary RF delivery at suboptimal site where a damage of the His-purkinje system might ensue. Pace mapping may help to differentiate a true distal Mahaim connection from early activated bystander Purkinje fiber. In conclusion when an atrio-fascicular Mahaim fiber must be ablated on the ventricular side, pace mapping may be useful in order to reduce the number of RF pulses needed to eliminate the distal insertion of the AP.

## Figures and Tables

**Figure 3:**